# A Trip to the Panama-Pacific Dental Congress

**Published:** 1916-01

**Authors:** W. Claude Adams

**Affiliations:** Portland, Ore.


### A TRIP TO THE PANAMA-PACIFIC DENTAL CONGRESS.

BY W. CLAUDE ADAMS, PORTLAND, ORE.

In point of attendance and in range of subjects covered, this congress of international scope was the most important in the annals of dentistry. Over two hundred original essays were presented and more than three hundred clinics were in operation. Twenty-six nations were represented, and the estimated attendance was two thousand. The sessions were held in San Francisco's new million-dollar auditorium, which has housed over eight hundred conventions since the beginning of the fair.

It was impossible for one to take in all papers and clinics, as there were ten sections in session at the same time. So great was the number and variety of subjects treated in the ten days' program that it was difficult to
value.
choose the part which would be of the most interest and

Among the most interesting features of the congress was the report of the work accomplished by the National Dental Research Commission, Dr. Weston A. Price, of Cleveland, chairman. The bureau has made startling discoveries concerning the action of bacteria, which will be of inestimable value to the medical as well as the dental profession.

To Dr. Weston A. Price, with the first moving pictures ever made showing the circulation of the blood, belongs the credit for creating the most profound sensation that marked the congress. Bacteria were shown whirling through the blood stream, affording a visual demonstration of the manner in which infections are swiftly carried from place to place in the body. The pictures were taken very much enlarged.

Of deep interest was the set showing the actual process of formation of an embolus, or blood clot, in the stomach of a frog. The demonstration came in the course of a paper prepared by Dr. Price on mouth infections. Dr. Price condemned the careless and slovenly methods of treating root canals, especially the use of any formalin preparation or mumifying paste in filling root canals. Over-medication is the cause of a great deal of root infection which goes out into the blood stream.

He also decried the wholesale extraction of teeth which ought to be saved. Since the medical men have learned that a diseased mouth may be the focus of infection, they have ruthlessly ordered the extraction of teeth which could, by proper treatment, be restored to normal function. The X-ray as an aid to diagnosis in this respect is indispensable. The loss of teeth weakens the patient, in that the stomach and whole body suffer.

Scientific investigation, by the Research Commission regarding the amboeba as a probable cause of pyorrhea and emetine as a cure, resulted in a defeat of the theory.

Dr. Price enumerated about seven good and logical reasons why the endamoeba is not the cause of pyorrhea, and put it up to the audience whether to take the judgment of a few medical men who have spent a very short time in the study of mouth infections or that of dental bacteriologists who have spent years in research work.

Dr. T. B. Hartzell, of Minneapolis, one of America's foremost authorities on pyorrhea, gave lectures on ''Special Researches in Mouth Infections'' and ''Regeneration of Bone,'' illustrated with slides showing method of proper instrumentation for pyorrhea, and patients before and after treatment. He discussed the endamoeba theory, agreeing with Dr. Price; in fact, the consensus of the congress was that of decided opposition to this theory.

Conductive anaesthesia or nerve-blocking was the subject of two lectures and several clinics given by Dr. A. E. Smith, of Cleveland. The nerve-blocking is accomplished by means of injections of novocain-suprarenin directly into the nerve and by infiltration the operation producing no shock whatever. Dr. Smith demonstrated the fact that it is possible to operate on one-half of the jaw with but one injection.

Some of the achievements in the realm of modern surgery were shown in the skillful operations for cleft-palate and hare-lip performed at the hospitals by Dr. Truman W. Brophy, of Chicago, chairman of the section of oral surgery, Dr. Chas. H. Oakman, of Detroit, and Dr. W. H. G. Logan, of Chicago.

Dr. John S. Marshall, who was in charge of the section of operative dentistry, read a paper which was worthy of serious consideration on the subject, ''Acidimetric Study of the Saliva and its Relation to Diet and Caries.''

In no previous convention has there been so much time devoted to discussion of artificial dentures. This is due to the fact that Dr. J. Leon Williams, of London, has evolved the wonderful system of Trubyte teeth, the esthetic value of which has been recognized. The subject of Dr. Williams' lecture was "Some Fundamental Things in Dental Prothesis."

Dr. Oscar A. Strauss, of Milwaukee, read a paper on "Radium Treatment in Carcinoma of the Mouth."

The almost universal consumption of candy among children is one of the chief causes of mouth infections and dental decay, according to Dr. H. L. Howe, of Boston. He added:

"The candy habit is an important cause toward undermining the health of American children today. In our cities there is a shop for the dispensing of cheap candies opposite almost every school house. If the candy traffic could be regulated as the liquor traffic is, it would be a source of good."

Dr. J. V. Conzett, of Dubuque, Iowa, hopes to see the time when dentists will be compelled to take an examination every two years to prove their efficiency.

It was an agreeable surprise not to have a "Brother Bill" show up on the program with a paper on the business side of dentistry.

The one thing about the congress that impressed me most of all was the earnestness with which the men applied themselves to the work of the convention. It was apparent that they were there to get all the knowledge possible—to broaden their vision.

The crowds swarming around the clinics reminded one of a huge hive of bees without one drone among them.

While America is recognized as leading the world in dental achievements, we are not the only country that is making strides. Reports from Asia, Africa, North and

South America and even the islands of the sea convince us that they, too, are alert.

The delegates from Guatemala came as representatives of their government to give particular attention to the work of dental clinics and mouth hygiene in the schools.

The Japanese delegates were all from the Tokio Dental College, which is a very large school with a student body exceeding that of many of our own colleges. An exhibit was shown giving an idea of the progress of dentistry in Japan. A wooden denture was among the interesting features of this exhibit. It was a primitive method used in Japan over forty years ago, but was apparently very serviceable, ivory being used for the front teeth and small shot imbedded in the wood for the molars. A primitive tooth-drilling instrument was also shown. It was simply a long stick with a bit of sharpened steel at one end and was twirled by means of a string.

The San Francisco daily papers were very generous in reporting the convention, giving space in nearly every issue. But the newspapers still persist in ridiculing the dental profession by such prominent headlines as "Knights of the Forceps" and "Mouth Plumbers," which is pathetic rather than amusing, for it only displays their ignorance of the true mission of the dentists and shows how far behind the times the general public is; as the majority of people depend on newspapers for their information.

I fear it will take years and years of preaching to bring the people to the point of realizing the rightful place the dental profession holds as guardian of their health.

Gloom was thrown over the convention when news was received from Chicago of the death of Dr. G. V. Black, whose title among dentists is that of "Father of Dentistry." To Dr. Black belongs the credit of having revolutionized operative and scientific dentistry.

The congress passed resolutions of sympathy and a copy of the same was forwarded to the family of Dr. Black.

In the room where the G. V. Black Club was conducting clinics in gold foil operations, the picture of Dr. Black was draped in crepe.

Resolutions commending George Eastman, of Rochester, N. Y., for his gift of $1,200,000 for a free dental dispensary at Rochester were unanimously passed by the house of delegates.

Commendation is due the officers on the success of the congress and the splendid management of so vast an undertaking.

The program committee deserves special mention for the preparation of the beautiful souvenir program, which was indeed a work of art.

Among the social features provided for visiting dentists were the reception and grand ball, a banquet and fraternity ball, a boxing tournament which was a stag affair, a luncheon given to the women delegates by the women dentists of San Francisco and an automobile trip given members of the congress and their ladies by the Alameda Society. The ride covered all points of interest in and around Oakland, Berkeley and Alameda, including a visit to the Greek theater and a view of the University grounds.—*Northwest Journal of Dentistry.*

